# Nanopathways modulating postoperative cognitive dysfunction: extracellular vesicles

**DOI:** 10.3389/fcell.2025.1613378

**Published:** 2025-06-30

**Authors:** Yunmeng Zhang, Zengsheng Yin, Zhiyong Zou, Shangzhi Feng, Huayang Xu

**Affiliations:** ^1^ Department of Anesthesiology, Jiujiang College Hospital, Jiujiang, Jiangxi, China; ^2^ Department of Urology, Jiujiang University Clinic College/Hospital, Jiujiang, Jiangxi, China

**Keywords:** postoperative cognitive dysfunction, extracellular vesicle, nano-targeted therapy, neuroinflammation, neurorestoration, cell-free therapeutic pathway

## Abstract

Postoperative cognitive dysfunction is a common central nervous system complication after general anesthesia in the elderly, and when it occurs, it will seriously affect the patient’s postoperative recovery and quality of life, which puts elderly postoperative general anesthesia patients at an extremely uncertain risk of postoperative psychiatric disorders or even death. It is currently believed that neuronal damage and inflammatory response due to cerebral ischemia/reperfusion injury induced by transient or repeated global cerebral ischemia during surgery are the key mechanisms for the development of postoperative cognitive dysfunction. Therefore, repairing postoperative neuronal damage and reducing neuroinflammatory responses may be an effective means of early intervention for postoperative cognitive dysfunction. Extracellular vesicles, a therapeutic tool with clear advantages in regenerative medicine, have been suggested as potential nanopathways to modulate postoperative cognitive dysfunction due to their pro-regenerative, pro-repair, and influence on immune responses. In this paper, we will summarize studies related to extracellular vesicles in the treatment of postoperative cognitive dysfunction and discuss the potential function of extracellular vesicles in nerve repair and inhibition of acute neurological inflammation, which will expand the therapeutic strategies for postoperative cognitive dysfunction and may represent the development of novel cell-free therapeutic pathways for modulating postoperative cognitive dysfunction.

## Introduction

Postoperative cognitive dysfunction (POCD) is a common complication of general anesthesia surgery, manifesting as agitation, confusion, and loss of learning and memory abilities, with memory loss being the most prominent feature (Liu et al., 2023). This condition can persist for months or even years, and untimely and inappropriate interventions may lead to dementia or even risk of death, which severely hampers postoperative recovery in elderly patients and makes the perioperative period extremely precarious for elderly patients undergoing general anesthesia surgical procedures. The occurrence of POCD is associated with a variety of factors, including age, education, preoperative complications, type of anesthesia, degree of surgical trauma, type of surgery, and postoperative Pain (Oriby et al., 2023; Ding et al., 2021). Large-scale clinical studies have shown that POCD occurs in about 30% of cases at 7 days postoperatively and in about 10% of cases at 3 months postoperatively (Yang et al., 2022). In terms of type of surgery, patients undergoing cardiovascular and orthopedic surgeries had the highest prevalence of postoperative cognitive deficits, at about 40% (Progress of research in postoperative cognitive dysfunction in cardiac surgery patients: a review article), while non-cardiovascular surgeries accounted for about 23.8% (Silva et al., 2021). POCD can seriously affect patients’ quality of life and increase the difficulty and burden of care, while soaring hospitalization costs as well as increased risk of mortality and dementia contribute to the adverse consequences of POCD. Therefore, early detection, diagnosis and intervention of POCD will effectively improve the quality of life of elderly surgical patients. However, the mechanism of POCD is unclear, and uniform clinical criteria for diagnosing POCD are still lacking. Therefore, it is imperative to find reliable and convenient clinical biomarkers. Currently, it is believed that the pathogenesis of POCD mainly focuses on the dysregulation of neuronal damage, neuroinflammation, oxidative stress, impairment of synaptic structural plasticity, and lack of neurotrophic support (Zhao et al., 2024; Lin et al., 2020). The most researched and considered major factors are neuronal damage and neuroinflammation, which are in fact considered to be the end result of other factors that ultimately trigger the onset and progression of POCD (Lin et al., 2020).

With the development of nanoscience and technology, extremely microscopic biomaterials are considered to be helpful in solving neurodegenerative disorders. With the regenerative repair, immune interference, and its unique cell affinity, targeting, and nano-delivery platform effects of extracellular vesicles, extracellular vesicles have been recognized as potential nanobio-pathways that can prevent and treat related neurological disorders (e.g., Alzheimer’s disease, Parkinson’s disease). For example, extracellular vesicles deliver therapeutic genetic material, such as miRNAs and proteins, which can exert neuroprotective effects and reduce cognitive impairment. As mediators of intercellular communication, extracellular vesicles are implicated in amyloid degradation, brain clearance, and intercellular diffusion of tau, and neuronal damage can be repaired by improving neuronal apoptosis through specific modifications of extracellular vesicles. The stem cell-derived extracellular vesicles also showed a strong regeneration-promoting function in brain injury due to their regenerative repair function (Hu et al., 2020). As far as neuroinflammation is concerned, extracellular vesicles in postoperative patients can act as a proactive mediator of postoperative cognitive dysfunction, and interfering with the development of these extracellular vesicles can help to impede and ameliorate COPD, e.g., treatment with GW4869 (an extracellular vesicle blocking agent) can be effective in preserving postoperative cognitive function by inhibiting the release of extracellular vesicles. In addition, extracellular vesicles from a variety of cellular sources also exhibit significant immunosuppressive functions, e.g., extracellular vesicles from mesenchymal stem cells reduce neuroinflammation (Cabrera-Pastor, 2024). Thus, extracellular vesicles may be a potential new modality for the prevention and treatment of postoperative cognitive dysfunction. Synthesizing the current studies and proposing the potential mechanism of extracellular vesicles for the treatment of POCD and preoperative medication pathway will greatly expand the prevention and treatment strategies for postoperative cognitive function.

## Postoperative cognitive dysfunction

In November 2018, the first international definition of perioperative neurocognitive disorder (PND) was proposed (Evered et al., 2018). Traditionally POCD, postoperative delirium, and preexisting cognitive impairment have all been defined as PND, while also emphasizing that cognitive deficits are associated with the entire perioperative period (Kong et al., 2022). With the rapid development of medical technology, the proportion of elderly patients undergoing surgery continues to rise, and the incidence of POCD has gradually increased, POCD has become a research hotspot in perioperative medicine (Liu and Leung, 2000). Studies have found that the overall incidence of POCD in older postoperative patients is approximately 25.8% within 1 week, and approximately 30%–60% of POCD occurs with delirium or cognitive dysfunction within 1 week of cardiovascular surgery. Previously, it was thought that all forms of cognitive impairment after anesthesia could be referred to as POCD, including POD, but more recently it has been argued that POCD is different from POD because POD is accompanied by altered consciousness, whereas POCD is not (H et al., 2017). Perioperative neurocognitive disorders in humans can lead to significant complications in the lives of patients, and patients with POCD will experience disturbances in consciousness, cognition, orientation, thinking, memory, or sleep, as well as social impairments after anesthesia and surgery. Patients with POCD often exhibit anxiety, confusion, and a loss of learning and memory abilities. This may increase the risk of developing different brain dysfunctions, including Alzheimer’s disease, long-term cognitive decline, and dementia, making POCD a highly worrisome condition, especially in the elderly population (Needham et al., 2017; Berger et al., 2019; Fong et al., 2021). Postoperative cognitive dysfunction can be triggered and exacerbated by a variety of factors, and the occurrence of postoperative cognitive dysfunction will seriously reduce postoperative recovery and lead to unavoidable medical disputes, but the relevant treatment methods are still very limited, and it is still necessary to find rapid and effective methods to prevent and control postoperative cognitive dysfunction, however, all the preventive and curative measures should be based on the premise that the etiology of the disease is clearly defined and the mechanism of the disease is clearly defined.

### Etiology and mechanisms

Factors closely associated with POCD include perioperative factors such as age at surgery, preoperative education, basal status, duration of surgery, type of surgery, type of anesthesia, and intraoperative hemodynamic changes, as well as postoperative factors such as infections, pain, and sleep disorders (Le et al., 2014; Monk et al., 2008) (Table 1). Of these, advanced age was identified as the only risk factor for long-term POCD (>3 months postoperatively) (Le et al., 2014; Monk et al., 2008; Moller et al., 1998). Although surgery is effective in treating a wide range of diseases, it can also lead to postoperative cognitive deficits, which may be associated with intraoperative and postoperative neuroinflammation induced by a variety of factors, neuronal injury, microecological dysregulation, defective autophagy in neuronal cells, and immune dysregulation (Huang et al., 2025). Different types of surgeries may also have an effect; older patients undergoing orthopedic and cardiovascular surgeries are more likely to develop POCD, possibly because such surgeries are more likely to cause widespread inflammation and neurologic changes (Wei et al., 2017; Hovens et al., 2016). Studies have found that POCD frequently occurs after coronary artery bypass grafting (CABG), and that the incidence of early cognitive dysfunction after CABG is high, at about 30%–60% (Boodhwani et al., 2006; Liu et al., 2009; Bishawi et al., 2018). For example, it has been found that transient or repeated cerebral ischemia during surgery can induce cerebral ischemia/reperfusion injury which in turn leads to neuronal damage and neuroinflammation, which is considered to be the key pathogenesis of POCD (van Harten et al., 2012; Lu et al., 2022). And massive intraoperative blood loss can lead to circulatory fluctuations and insufficient blood supply to the brain, which can affect oxygen supply, brain cell metabolism, and postoperative cognitive function (Zhang et al., 2019). For postoperative factors, postoperative pain and sleep disturbances are thought to be associated with the development of POCD, e.g., effective analgesia and adequate sleep may reduce the incidence of POCD (Wang et al., 2014). Although the mechanism is still unclear, it may involve the body’s stress response due to pain to trigger an anxious and depressed state, and the sleep state can effectively promote the removal of metabolic waste. In addition, maintaining a positive preoperative state of mind may also help reduce postoperative delirium (POD) and promote postoperative cognitive improvement (Hudetz et al., 2010). It is evident that POCD is not only related to neurological disorders but also to psychiatric disorders. However, the relationship between anesthetic drugs and depth of anesthesia and the incidence of POCD is still controversial (Hou et al., 2018; Lu et al., 2018; Farag et al., 2006). Indeed, the etiology of POCD varies depending on the purpose of the study, which depends on the population included, assessment criteria, interventions, type of surgery and other factors. For example, studies have found that the fewer years of education and knowledge base a patient has, the higher the incidence of POCD, but this still needs to take into account geographic and gender differences (Scott et al., 2017).

**TABLE 1:** Risks of POCD and interventions.

Type of surgery	Risk factors	Intervention	References
Gastrointestinal Surgery	advanced age	Cognitive Training	Saleh et al. (2015)
Cesarean section	Surgical Stress	Stellate ganglion block	Deng et al. (2023)
Solid tumor resection	Educational background	Improvement of cognitive reserve	Kainz et al. (2023)
Thoracic surgery	Sleep disorders	Treatment of preoperative sleep disorders	Li et al. (2021a)
Tibial Fracture Surgery	Postoperative decline in mitochondrial function	Drugs to improve mitochondrial function	He et al. (2022)
Carotid artery exposure	Postoperative neuroinflammation, oxidative stress and synaptic dysfunction	Transcranial near infrared laser irradiation	Zhong et al. (2022)
Apical hepatectomy	Postoperative mitochondrial dysfunction	Dexmedetomidine to reduce mitochondrial damage	Sun et al. (2023)
Lobectomy	Pain	Dexmedetomidine preoperative sedation and analgesia	Shi et al. (2020)
Partial hepatectomy	Postoperative neuroinflammation	Dexmedetomidine inhibits hippocampal inflammation	Chen et al. (2019)
Hip replacement	Pain	Oxycodone for postoperative paroxysms	Gan et al. (2020)
Heart Surgery	Postoperative systemic inflammation	Ketamine to suppress systemic inflammation	Hudetz et al. (2009)
Partial hepatectomy	Postoperative neuroinflammation	Electroacupuncture inhibits neuroinflammation	Yuan et al. (2014)
Inhalation Anesthesia	Anesthetic Drug Toxicity	Nimodipine reverses sevoflurane toxicity	Cui et al. (2018)
Inhalation Anesthesia Surgery	Anesthesia Drug Toxicity	Cistanche extract inhibits neuroinflammation	Peng et al. (2020)
Carotid Artery Exposure	Postoperative neuroinflammation	Amantadine maintains neurotrophic	Zhong et al. (2020)

Sleep disorders are thought to be an important contributor to POCD, with chronic reductions in sleep duration or complete lack of sleep leading to cognitive deficits. For example, maintaining sleep duration at 4–6 h per night can lead to neurobehavioral deficits during waking hours. In contrast, patients’ reduced daytime strength, functional limitations, and emotional vulnerability after surgery will negatively affect postoperative cognitive recovery (Xie et al., 2013). In fact, postoperative cognitive dysfunction (POCD) and AD have similar pathogenesis (Hu et al., 2010).

During sleep, the interstitial volume of the cerebral cortex can increase by up to 60%, resulting in increased convection of ISF and CSF, which is effective in clearing Aβ. Pathological tau accumulation is more strongly associated with cognitive decline than Aβ, and sleep disorders can lead to hyperphosphorylation and aggregation of tau proteins, which in turn can lead to the formation of neural protofibrillar tangles, neurinflammatory plaques, and other structures (Brier et al., 2016). Sleep disturbances are also associated with hemodynamic instability and changes in pulmonary ventilation, which increase the incidence of postoperative fatigue, anxiety, depression, and pain sensitivity and thus decrease the rate of recovery from postoperative cognitive deficits, which leads to prolonged hospital stays. Postoperative pain can also severely affect postoperative cognitive recovery. Postoperative pain is a complex series of pathophysiological responses to surgical trauma involving cellular inflammation, nerve damage, and synaptic remodeling, which are closely related to the development of postoperative cognitive dysfunction. The hippocampus plays a key role in regulating emotion, learning and memory formation during cognitive processes, and postoperative pain significantly induces cellular stress and inflammatory responses and induces the release of inflammatory cytokines in the hippocampus, which in turn impairs hippocampal function (Chen et al., 2020; Conner et al., 2009). In addition, the toxicity of anesthetic drugs has been linked to postoperative cognitive dysfunction, and new evidence from experimental models in humans and rodents *in vivo* suggests neurobiological changes and lifelong cognitive deficits following exposure to anesthetic agents. Studies have shown that exposure to isoproterenol and sevoflurane causes cognitive deficits in mice (Lv et al., 2017). Anesthetics cause changes in the blood-brain barrier and cerebrovascular system by altering neuronal networks that may also contribute to long-term neurocognitive dysfunction in the brain. Since both intravenous and inhalational anesthetics currently in use produce anesthetic and sedative effects by binding to GABA receptors and/or N-methyl-D-aspartate receptors (NMDA, a subtype of glutamate receptors), due to these mechanisms of their use, all of these drugs may contribute to the development of neurocognitive deficits in the postoperative period (Orser and Wang, 2019).

**FIGURE 1:**
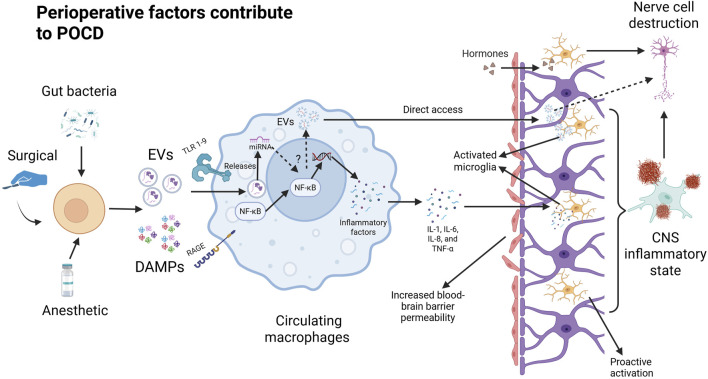
Perioperative factors contribute to POCD.

Postoperative neuroinflammation, systemic immune activation, and oxidative stress in cells have been found to be key mechanisms in the development of postoperative cognitive dysfunction (Cibelli et al., 2010) (Figure 1). In terms of postoperative systemic inflammation, surgical trauma induces the release of proinflammatory cytokines and chemokines at the site of tissue injury, triggering a localized inflammatory response. These inflammatory mediators can activate peripheral immune cells and disrupt the tight junctions of the blood-brain barrier, and these peripheral immune cells (macrophages and monocytes) can then infiltrate the nervous system to and further contribute to the neuroinflammatory cascade. In the CNS, microglia play a critical role as immune cells, and their dysfunction can lead to neuroinflammation and negatively impact overall health. Microglia help reduce neuroinflammation by phagocytizing proteins such as Aβ, tau, and α-synuclein (Loh et al., 2024). Because central microglia are particularly sensitive to pathological injury and can respond immediately to infection, inflammation, and neurological injury, this inflammatory state can activate microglia to polarize toward inflammation (M1 phenotype) and lead to further release of proinflammatory cytokines and chemokines, thereby triggering a cascade of neuroinflammatory responses (Li et al., 2010). In contrast, microglia polarization responses have a huge impact on neuronal activity and synaptic plasticity in the hippocampus, ultimately affecting cognitive function. For example, a systematic evaluation of animal experiments has shown that microglia activation is associated with elevated levels of IL-1β, TNF-α, and Toll-like receptors (activated microglia release cytokines and chemokines, such as IL-1β, IL-6, and TNF-α), and may lead to delirium (Hoogland et al., 2015; Author Anonymous, 2024). Additional studies have also confirmed that the inflammatory response plays a key role in the onset and progression of POCD, and that activation of the systemic inflammatory cascade response will result in systemic cytokine dysregulation, which is thought to be an important factor in neurodegeneration and subsequent cognitive impairment in delirium (Momen-Heravi et al., 2014). This is because the elderly are often in a state of chronic low-grade inflammation and immunocompromise, which makes them more susceptible to the inflammatory effects of infection and trauma. When an elderly patient is subjected to major trauma (e.g., a serious injury or fracture), the systemic acute inflammatory response can be triggered rapidly and involves the release of cytokines and the aggregation of inflammatory cells, which is followed by a high likelihood of POCD after undergoing anesthesia and major surgery. POCD and major surgical procedures or anesthetic drugs can elicit a secondary inflammatory response that can exacerbate the severity of the inflammatory response. Such secondary inflammatory events can lead to immune dysregulation, disruption of cellular function, and negative effects on multiple systems, including an increased risk of POCD (Qin et al., 2024). In addition, this chronic inflammatory state can also lead to mild neuroinflammation characterized by upregulation of central proinflammatory cytokines and increased numbers of microglia (Li et al., 2022). In addition to aging itself, chronic diseases associated with aging, such as obesity, diabetes, cardiovascular disease, *etc.*, can also lead to the body in a state of “chronic low-grade inflammation” (Kawai et al., 2021). In fact, mice that underwent major surgery after a one-time inflammation showed a further decline in physical activity that also led to more severe cognitive impairment.

In addition to the inflammatory response, POCD has been found to be involved in a variety of pathological processes such as neuroinflammation, mitochondrial dysfunction, oxidative stress, blood-brain barrier damage, impaired neurotrophic support, and synaptic injury and has been well-studied. Evidence suggests that these associated molecular pathways may increase perioperative complications and mortality (Lin et al., 2020). However, at present, inflammatory response and nerve damage are considered to be the more recognized key mechanisms of postoperative POCD, and many therapeutic options are mainly based on this mechanism and have achieved some therapeutic effects, although the current therapeutic options are extremely limited. Therefore, summarizing and describing the potential new nanoscale solutions by inhibiting inflammation and repairing the most nerve damage will greatly expand the treatment options for postoperative POCD and may lead to new breakthroughs in nanotherapeutics.

Perioperative factors lead to the release of DaMPs from damaged cells that are recognized by the corresponding receptors in circulating macrophages. Binding to TLR and RAGE at the macrophage membrane triggers the NF-κB pathway in the cytoplasm. Degradation by phosphorylation and ubiquitination exposed the nuclear translocation site in NF-κB and induced nuclear translocation of NF-κB. In the nucleus, NF-κB regulates the expression of proinflammatory genes and promotes the cellular secretion of inflammatory factors (IL-1, IL-6, IL-8, and TNF-α). Pro-inflammatory factors disrupt the tight junctions of the blood-brain barrier after which inflammatory factors, danger signaling molecules, and peripheral immune cells enter the brain through the damaged blood-brain barrier to further activate microglia. At the same time, the damaged cells may also release specific extracellular vesicles that prompt peripheral immune cells (including circulating macrophages) to produce inflammatory factors or extracellular vesicles that can directly damage neuronal cells through the blood-brain barrier.

### Status of treatment

POCD is a complication of the central nervous system characterized by intellectual disability, anxiety, personality changes, and impaired memory that manifests as neurocognitive deficits, affecting many elderly patients recovering from surgical procedures, where declines in memory, attention, and executive functioning can significantly affect quality of life and increase the risk of long-term cognitive impairment. Currently, the treatment of POCD relies on medication and rehabilitation such as acupuncture (Table1), focusing on reducing inflammatory responses and maintaining neurotransmitter balance, such as the narcotic drugs dexmedetomidine, etomidate, and ketamine; the anti-inflammatory drugs parecoxib and cyclooxygenase-2; and the antipsychotic drugs galantamine and sarcosine methyl, haloperidol, and so on (Wang et al., 2021). Acupuncture, on the other hand, is mainly for electro-acupuncture stimulation of acupoints to reduce the postoperative stress response thereby alleviating the degree of nerve damage (Ho et al., 2020). In contrast, non-pharmacological options such as preoperative and postoperative cognitive training are usually preferred for the treatment of POCD in the clinical context (Butz et al., 2022; Saleh et al., 2015). This is due to the fact that various medications are not developed for POCD, which can have numerous adverse effects on patients. For example, postoperative use of non-steroidal anti-inflammatory drugs for pain relief may lead to gastric bleeding and kidney damage and is not suitable for long-term use; anesthetic drugs are not suitable for patients with chronic diseases such as heart, lungs and brain, especially for the elderly. Although some clinical reports suggest that the use of lidocaine (a local anesthetic) or parecoxib (a nonsteroidal anti-inflammatory drug) may reduce the incidence of POCD (Ho et al., 2024). Even though certain medications, such as cholinesterase inhibitors, N-methyl-d-aspartate receptor antagonists, and antipsychotics, have been used in clinical practice to improve cognitive function, there are no specific medications approved by the U.S. Food and Drug Administration for the treatment of POCD (Gao et al., 2024). Therefore, there is a need to study and develop effective control programs to help patients regain cognitive function at different stages of the postoperative period. Although anti-inflammation, inhibition of microglia activation and improvement of cerebral microcirculation are potential coping strategies for POCD, neither pharmacologic nor non-pharmacologic approaches have achieved satisfactory clinical results (Lin et al., 2020). There is an urgent need to explore reliable detection and treatment methods, and the international community has called for systematic research on POCD.

## Extracellular vesicles modulate postoperative cognitive dysfunction

### Modulation of neuroinflammatory responses

Inflammatory responses play a key role in the development and progression of a variety of human diseases, including autoimmune, neurodegenerative, and other inflammatory disorders, and have also been recognized as a major feature of several neurological disease-related pathological conditions, such as Alzheimer’s disease and postoperative cognitive dysfunction. Neuroinflammation has been identified as a key mechanism contributing to cognitive impairment, and a large body of clinical evidence highlights the significant impact of immune dysfunction on cognitive decline. Surgical activation of immune cells in the immune system (e.g., macrophages and neutrophils) infiltrates neural tissue through the disrupted blood-brain barrier and thus leads to neuroinflammation. Although the inflammatory response to tissue damage or destruction removes harmful substances and damaged tissue. However, excessive nervous system inflammation can lead to neurotoxicity and cell death. In turn, damaged neurons release Aβ to cause inflammation, thus initiating a vicious cycle of continuous Aβ release, similar to Alzheimer’s disease (Krstic and Knuesel, 2013). In addition, central inflammation affects neurotrophic factor levels, which disrupts synaptic plasticity and leads to decreased nerve regeneration. The resulting excessive inflammation in the brain can lead to postoperative cognitive decline (Solas et al., 2017). Extracellular vesicles are important carriers that mediate the transfer of active substances and genetic information between cells. It was found that the preoperative message carried by exosomes released by peripheral monocytes/macrophages has been altered in older patients who have experienced major illness or trauma (Qin et al., 2024). Another study reported that extracellular vesicles released by macrophages, when injected *via* jugular vein, aggregated more prominently in the brains of mice with the presence of central nervous system inflammation than in normal mice (Dong et al., 2021). In contrast, postoperative mice treated with GW4869 had cognitive function and blood-brain barrier function closer to those of normal mice, and significant reductions in the levels of inflammatory factors in the peripheral blood and central hippocampus of the mice. Furthermore, injection of macrophage-released exosomes into healthy mice induced inflammation, hippocampal damage, and cognitive deficits, which were significantly alleviated by treatment with GW4869 (Qin et al., 2024). Thus, extracellular vesicles are likely to be important mediators in mediating the postoperative peripheral immune response to induce central inflammation. Twenty-five EVs associated with M1-type microglia have been reported to modulate neuronal inflammation involved in cognitive impairment (Medders et al., 2010; Zhou et al., 2016). For example, EVs released from M1-microglia overexpressing IL-1R1 can promote POCD development by modulating neuronal inflammation. Another study reported that extracellular vesicles released by macrophages, when injected *via* jugular vein, aggregated more prominently in the brains of mice with the presence of central nervous system inflammation than in normal mice (Dong et al., 2021). Extracellular vesicles may help deliver therapeutic genetic material, such as miRNAs and proteins, to exert neuroprotective effects and reduce cognitive impairment. For example, in an animal model of persistent pulmonary hypertension, extracellular vesicles inhibit macrophage infiltration and the release of proinflammatory mediators through miRNA-mediated decreases in the levels of monocyte chemotactic proteins and mitogens, which effectively control the inflammatory response caused by the surgery and thus reduce the occurrence of POCD. The release of extracellular vesicles containing miR-124-3p from microglial cells also exerts a protective effect against cognitive deficits by attenuating the inflammatory polarization process of hippocampal microglia in aged mice (Xin et al., 2017). Embryonic stem cell-derived extracellular vesicles also alleviate long-term diabetes-induced chronic inflammation in neural tissue to improve postoperative cognitive dysfunction in mice (Lang et al., 2023). Therefore, interfering with the generation of extracellular vesicles as mediators of postoperative cognitive dysfunction and special cellular sources with anti-inflammatory functions or utilizing their anti-inflammatory functions may be a potential pathway to ameliorate postoperative cognitive dysfunction.

### Promotes neural tissue survival

The regenerative repair function of extracellular vesicles of specific cellular origin has been extensively studied, and their neurorestorative function is gradually being recognized. In postoperative cognitive dysfunction, neuronal damage is the direct cause of the onset of cognitive impairment (Lang et al., 2023). Repairing and promoting neuronal survival may help to inhibit the onset and slow the severity of postoperative cognitive dysfunction. Among them, stem cell-derived extracellular vesicles are most widely used in neural cell regeneration and repair therapy, mainly due to their high self-renewal capacity, high plasticity, low immunogenicity and effective cellular therapeutic efficacy (Andrzejewska et al., 2021). First, exogenous extracellular vesicles can target brain cells across the blood-brain barrier (Wang et al., 2020). Second, stem cell-derived extracellular vesicles exhibit robust neurogenesis and cognitive recycling (Lang et al., 2023). For example, embryonic stem cell-derived extracellular vesicles have shown potent regenerative functions in brain injury, and have also shown beneficial effects in promoting angiogenesis, stem cell proliferation and differentiation (Hu et al., 2020; Hu et al., 2015). In addition, extracellular vesicles that maintain neural repair and regeneration are also present in the central system. miR-124-3p-containing extracellular vesicles released by exogenous microglia in the hippocampus improve cognitive function by repairing axonal demyelination and overexpressing neurotrophic factors. Previous studies have demonstrated that microglia in the hippocampus continuously synthesize, assemble and secrete various types of extracellular vesicles into synapses to regulate synaptic plasticity and neural activity (Li Q. et al., 2021; E et al., 2022). Extracellular vesicles in the hippocampal microenvironment contain miR-124-3p that can also alter the expression of various neurotrophic factors that are essential for nerve growth. For example, BDNF promotes neuronal growth and differentiation and enhances synaptic plasticity (Huang et al., 2018; Ge et al., 2020). Since miRNA expression is associated with surgery-induced neuronal injury, extracellular vesicles carrying and transporting miRNAs may be important elements in neuronal survival or death. For example, knockdown of extracellular vesicles of miR-206 inhibits neuronal apoptosis after acute brain injury. It is well known that microglia polarization has a huge impact on neuronal activity and synaptic plasticity in the hippocampus, which can ultimately affect cognitive function. Previous studies have demonstrated that microglia in the hippocampus can secrete miRNA-containing extracellular vesicles into the microenvironment to regulate microglia polarization and neurodegeneration (Qian et al., 2022). For example, central hippocampal microglia contain miR-124-3p extracellular vesicles involved in the process of autophagy and apoptosis in neurons to ameliorate postoperative cognitive dysfunction. In contrast some substances in microglia extracellular vesicles damage neurons at a later stage, such as IL-1β, soluble toxic Aβ peptide, and caspase-1 (Kong et al., 2024). Since miRNAs are involved in multiple pathophysiological processes involved in POCD occurrence, interfering with the extracellular vesicles that transmit miRNAs may be a new target for POCD therapy by enhancing neuronal survival.

### Improvement of postoperative pain

Postoperative pain-induced cognitive impairment severely worsens the outcome of rehabilitation in elderly patients. Elderly patients are often sensitive to surgical trauma, stress, anesthesia, or pain, which greatly increases the incidence of postoperative cognitive impairment, especially after undergoing cardiovascular, orthopedic, or cerebrovascular surgery. Postoperative pain-induced cognitive deficits in elderly patients are becoming a pressing issue, and patients with cognitive deficits often experience severe impairments in social activities, learning, and memory, which are detrimental to the quality of recovery in elderly patients. Postoperative pain is associated with the development of POCD, and although the mechanisms involved are unclear, it may be related to the body’s stress response due to pain, which triggers a state of anxiety and depression. Several studies have confirmed that effective analgesia can reduce the incidence of POCD (Wang et al., 2014). For example, postoperative morphine analgesia inhibits the expression of proinflammatory factors, cell cycle protein D1, in the hippocampus and promotes the expression of anti-inflammatory factors in the central system (Kong et al., 2024). As extracellular vesicles derived from stem cells are also effective in relieving postoperative pain, the use of these extracellular vesicles may be a way to improve postoperative cognitive dysfunction. Abnormally activated microglia act as an innate central immune cell, which can exacerbate inflammatory pain by upregulating inflammatory factors. Extracellular vesicles derived from human umbilical cord mesenchymal stem cells have been shown to alleviate inflammatory neuropathic pain caused by microglia activation (Hua et al., 2022). Neuroinflammation is a common feature of most neurologic dysfunctions and is also strongly associated with postoperative pain. By reducing neuroinflammation it is also effective in reducing postoperative pain and ultimately facilitating recovery from postoperative cognitive dysfunction. For example, miR-124-3p expression in microglial extracellular vesicles was shown to be downregulated in the hippocampus of mice with postoperative pain. Protective effect of postoperative pain production against cognitive impairment by modulating inflammatory polarization of hippocampal microglia in aged mice can be reduced (Kong et al., 2024). In addition, various forms of extracellular vesicles have been shown to reduce signs of neuronal inflammation thereby alleviating neuropathic pain in animal models. For example, extracellular vesicles from synovial MSCs, human placental stem cells, dental pulp stem cells, and bone marrow MSCs have all demonstrated the ability to reduce inflammation to relieve pain in animal models (Shipman et al., 2024). Interestingly, self-metabolically regulated processes (autophagy, pyroptosis) in neuronal cells are also associated with neuropathic pain, with activation of astrocyte autophagy decreasing the pain level and inhibition of autophagy exacerbating the pain, regardless of whether neuropathic pain is at any stage of induction or maintenance. The activation of cellular pyroptosis also exacerbates neuroinflammation and thus neuropathic pain. In contrast, human umbilical cord mesenchymal stem cell-derived extracellular vesicles have been found to attenuate inflammatory neuropathic pain by enhancing autophagy and inhibiting cellular focal death. Therefore, pain alleviation and thus effective reduction of POCD by utilizing the inhibitory neuroinflammatory effects of extracellular vesicles may be a potential approach for the treatment of POCD.

### Reducing postoperative sleep disturbances

Postoperative sleep disorders are a common complication after major surgery, and patients usually present with a persistent decrease in the quality and duration of their sleep (Gögenur et al., 2008). Surgery is associated with sleep fragmentation and deprivation, as well as reduced rapid eye movement and slow wave sleep, which results in changes in postoperative brain function and increases the risk of postoperative cognitive decline, and prolonged reductions in postoperative sleep duration or complete sleep deprivation have been shown to result in cognitive deficits (Sipilä and Kalso, 2021). Therefore, early identification and intervention of sleep disorders may be effective in reducing postoperative cognitive dysfunction, ultimately improving prognosis and shortening hospital stay (Shokri-Kojori et al., 2018; Bah et al., 2019). In terms of mechanisms, postoperative sleep disturbances can lead to amyloid plaque accumulation, tau protein diffusion, increased neuroinflammation, and increased blood-brain barrier permeability leading to postoperative cognitive decline (Gu and Zhu, 2021). Modulation of these processes may be a pathway to reversing postoperative cognitive deficits. Extracellular vesicles are important mediators of the transfer of active substances and genetic information between cells, and are involved in the development of sleep disorders and their subsequent resultant cognitive deficits by increasing the formation of amyloid plaques, transmitting tau proteins, modulating neuroinflammation, and increasing the permeability of the blood-brain barrier (Gu and Zhu, 2021). For example, inhibition of total extracellular vesicular secretion secretion *in vivo* decreases AD-like pathological processes, but the mechanisms involved in this process still need to be refined (Dinkins et al., 2014). Plasma extracellular vesicles obtained from sleep-rhythm-disordered mice can act as a bridge between peripheral clock-control genes and central rhythms and transmit the effects of circadian rhythm disruption to target organs, thereby disrupting end-organ homeostasis (Khalyfa et al., 2017). In terms of direct action, extracellular vesicles may be proteins that directly regulate signaling pathways or regulate proteins involved in signaling pathways or key enzymes. However, the specific molecular mechanisms behind these processes are still debatable. With the advantage that extracellular vesicle intervention and mediated miRNA modulation can reduce neurotoxic proteins produced by sleep disorders, extracellular vesicles, a nano-delivery pathway, may in the future provide new insights into the pathomechanisms and treatment of POCD. An *in vitro* study, for example, found that extracellular vesicles derived from N2a cells enhance Aβ uptake into microglia *via* their surface sphingolipids and ultimately reduce amyloid plaque formation (Perez-Gonza et al., 2012; An et al., 2013). Under hypoxic conditions, extracellular vesicles will help prevent and treat abnormally elevated Aβ levels caused by sleep disorders. In conclusion, sleep disorders are likely to be involved in the pathogenesis of postoperative cognitive impairment and increase the risk of postoperative dementia. And due to the advantages of extracellular vesicles in terms of reduced immunogenicity and better targeting. Delivery of therapeutic biomolecules to target cells *via* extracellular vesicles to correct physiological dysfunction is a potential therapeutic strategy for treating brain disorders characterized by exogenous disturbances (e.g., surgery, trauma).

### Regulates the microecology of the body

Increased permeability of the blood-brain barrier in the postoperative period is the main pathologic mechanism of POCD (Wang et al., 2017). During surgery, the integrity of the blood-brain barrier (BBB) may be compromised, allowing harmful substances normally blocked by the barrier to enter the brain. This may lead to inflammation and brain cell damage, which ultimately induces the development of POCD (Keshavarz et al., 2021). Specifically, the entry of harmful substances triggers microglia activation, which in turn promotes the secretion of pro-inflammatory factors, which in turn exacerbates BBB damage, resulting in a vicious cycle of inflammation and neurodegeneration (Wang Y. et al., 2019). In fact, the factors associated with BBB permeability impairment are not only related to the gastrointestinal tract’s communication with brain centers. The importance of the microbiome-gut-brain axis in central system disorders has gained prominence over the past 2 decades (Iyaswamy et al., 2023). It has been shown that alterations in gut flora reduce expression of tight junction proteins, leading to BBB leakage (Luo et al., 2021; Wen et al., 2020). Thus, surgically induced intestinal mucosal injury impairs the intestinal mucosal barrier function, allowing toxic metabolites and bacteria to enter the somatic circulation, which in turn enters the central system through the compromised BBB (Yang et al., 2023). Surgical and anesthetic procedures may alter the composition of the gut microbiota. Indeed, the gut microbiota of older mice with postoperative cognitive dysfunction was significantly different from that of normal mice (Zhang Sh et al., 2023). Changes in the abundance of various bacterial species and their metabolites before and after surgery may be associated with postoperative cognitive impairment (Zhang et al., 2024; Ren et al., 2024; Tsigalou et al., 2023). For example, increased abundance of pro-inflammatory gut microbiota triggers and worsens the systemic inflammatory response, promotes neuronal cell injury and inhibitory processes on neuroautophagy, and influences anti-inflammatory extracellular vesicle production and circulation, which collectively play a role in the onset and progression of cognitive deficits in the postoperative period. Interestingly, secretion of extracellular vesicles by some specific beneficial intestinal flora can also effectively reverse this process, e.g., mucinophilic Ackermannia-derived extracellular vesicles can ameliorate intestinal I/R-induced POCD in a mouse model by maintaining intestinal and BBB integrity. Therefore, analyzing perioperative changes in extracellular vesicles released by gut flora and exploring interventions based on these alterations could provide a promising approach to the prevention and management of neurocognitive disorders.

Extracellular vesicles of bacterial origin are important mediators of communication between different bacterial colonies as well as between colonies and hosts. These vesicles are mainly composed of lipopolysaccharides, lipids and proteins (Díaz-Garrido et al., 2021). They play an important role in maintaining the integrity of the host intestinal mucosal barrier, supporting the normal function of the host immune system, and regulating substance metabolism (Díez-Sainz et al., 2022; Tan et al., 2022). Changes in bacterial flora produce different types of extracellular vesicles, and dysbiosis can lead to abnormalities in these vesicles, which can have a more variable impact on host cognitive function. In a preclinical study, it was demonstrated that transferring fecal microbes from older to younger mice increases the likelihood of cognitive deficits and that such vesicles cause hippocampal damage (Lee et al., 2020). In addition, postoperative cognitive deficits may also be associated with an increase in pro-inflammatory bacteria, such as extracellular vesicles from the intestinal flora of AD patients that activate GSK-3β proteins, induce tau protein phosphorylation, and enhance the secretion of inflammatory cytokines in the hippocampus (Wei et al., 2020). All these processes suggest that extracellular vesicles are involved in the gut-brain axis to induce the onset and progression of POCD. Therefore, by interfering with the occurrence and release of extracellular vesicles may be a potential way to regulate the organism’s microecology to effectively ameliorate POCD.

### Reduced neurotoxicity of anesthetics

Anesthetic neurotoxicity is defined as damage to the structure and function of the nervous system caused by exposure to anesthetic drugs (Campo et al., 2024). *In vivo* experimental models have shown neurobiological changes and lifelong cognitive deficits following exposure to anesthetics. It is possible that anesthetics interfere with the proliferation and differentiation of immature neurons and that neurogenic changes can be observed after exposure to different anesthetics (including isoproterenol, isoflurane, and sevoflurane) in young and adult brains (Fang et al., 2012). For inducing postoperative cognitive dysfunction, this may be due to the fact that anesthetics can induce changes in the blood-brain barrier and the cerebrovascular system by altering the neuronal network of the brain for long-term neurocognitive dysfunction (Zanghi and Jevtovic-Todorovic, 2017). As the most widely used anesthetics, exposure to isoproterenol and sevoflurane will often exacerbate postoperative cognitive impairment in patients (Lv et al., 2017). All currently used intravenous and inhaled general anesthetics produce anesthetic and sedative effects by binding to GABA receptors and/or N-methyl-D-aspartate receptors. This can lead to minor neurocognitive deficits in patients after surgery, such as postoperative delirium. First, this is because with the use of perioperative anesthetic drugs GABAA receptors are potentiated, which alters the opening of intracellular ion channels affecting the inflow and outflow of chloride ions leading to excessive neuronal inhibition that persists long after the drugs have been eliminated (Orser and Wang, 2019). Second, proinflammatory cytokines released during surgery also induce cell surface overexpression of extrasynaptic GABAA receptors. Both mechanisms may lead to permanent neurocognitive deficits in patients after surgery (Orser and Wang, 2019). Perioperative neurocognitive deficits may carry a serious risk of developing dementia, memory loss, loss of concentration and even death (Rudolph and Marcantonio, 2011). Therefore, intervening and ameliorating the neurotoxicity of anesthetics may be a necessary part of preventing POCD. Biomolecules that control adverse reactions to anesthetic drugs may help reduce the development of postoperative brain dysfunction, study finds. It was found that extracellular vesicles are capable of transporting active ingredients that represent the extracellular microenvironment, and that all cell types can communicate with each other through this pathway, exchanging molecules needed to maintain homeostasis. In this case, any alteration affecting each cell type can to a large extent be compensated by the other cell types, being able to provide the tissue with all the effective molecules needed to overcome the perturbation or to induce specific epigenetic modifications that allow the synthesis of molecules that restore the *in vivo* equilibrium or even prevent it from being altered (Campo et al., 2024). Of course, this does not preclude the transport of extracellular vesicles from providing the deleterious molecules needed to promote perturbation. In the *in vitro* circulation, extracellular vesicles can exert a protective effect against anesthetic-dependent adverse effects by transmitting overexpression of miR-34a and miR-124 in the central system. YRNA1 in circulating extracellular vesicles is significantly overexpressed in the postanesthetic state, and its upregulation correlates closely with the ability of the central system to compensate for anesthetic-induced inflammatory effects. In clinical practice, the incidence of POCD is higher in elderly patients anesthetized with sevoflurane (Ishii et al., 2016). This may be due to the higher utilization of sevoflurane compared to isoproterenol. It has been found that sevoflurane inhalation anesthesia promotes POCD in a dose-dependent manner, and that inhalation of sevoflurane can cause cognitive deficits and behavioral abnormalities in postoperative patients, accelerating the onset and progression of POCD (Cheng et al., 2018). This may be due to the fact that sevoflurane anesthesia produces associated extracellular vesicles involved in the activation of inflammatory and apoptotic neuronal pathways in the central system of the organism (Cui et al., 2018). For example, sevoflurane-induced associated extracellular vesicles can deliver miR-584-5p to promote the onset and progression of delirium by targeting BDNF to regulate Caspase3 and BDNF/TrkB signaling.

In addition, anesthetic drugs disrupt the balance of the gut microbiota through direct effects on the microbiota, potentially leading to dysbiosis, which in turn affects central neurocognitive function through the gut-brain axis (Iyaswamy et al., 2023; Liu et al., 2022). In addition, the effects of different anesthetic drugs and anesthetic methods on the microbiota may vary. Opioids can have significant effects on the gut microbiota that can lead to disruption of microbial and host metabolism. In addition, anesthesia-induced dysbiosis of the intestinal flora can lead to significant changes in the composition of extracellular vesicles and alter the ability of extracellular vesicles to enter the bloodstream, which ultimately affects systemic energy metabolism and thus the central system, which may be a potential mechanism for surgical damage to the intestinal microbial homeostasis leading to the development of POCD (Huh et al., 2019; Saeedi et al., 2019). Indeed, extracellular vesicles from different flora sources exert different effects on the host, which also include a protective effect on central cognitive functions (Diaz-Garrido et al., 2022). Therefore, implementation of appropriate preoperative and postoperative interventions to minimize damage to the microbiota ecosystem and repair disturbed microecology may be a future strategy for POCD treatment in the case of cognitive deficits caused by anesthetic drugs or surgery. Certainly, preventing or ameliorating the onset and progression of postoperative cognitive deficits by means of intervening on extracellular vesicles released after microecological disturbances, including inhibition of deleterious vesicles and promotion of protective vesicles (e.g., preoperative intestinal implantation of probiotic bacteria) may be an exploratory avenue of POCD nanotherapeutics.

## Advantages of extracellular vesicles in modulating postoperative cognitive dysfunction

### Biomolecular transfer function

There is growing evidence that circulating extracellular vesicles contain a large number of multifunctional RNAs (e.g., miRNAs, lncRNAs, circRNAs), which explains their key role in cellular communication. In this context, extracellular vesicles are considered important messengers that can transport a wide range of molecules at once, including cytokines, growth factors, various proteins, and even nucleic acids, thus determining their efficient delivery for transfer from 1 cell to another (Campo et al., 2024). These transported molecules may represent all the major components of the extracellular microenvironment, and all the biomolecular types that maintain homeostasis in the organism can communicate with each other through this mechanism, exchanging molecules required for correct homeostasis. Delivery of therapeutic RNA to target cells *via* extracellular vesicles to correct protein dysfunction is a potential therapeutic strategy for the treatment of brain disorders due to the low immunogenicity and good targeting properties of extracellular vesicles (Duan et al., 2021). For example, extracellular vesicle transferable miRNAs modulate the neuroinflammatory cascade response, Aβ production, and neuronal apoptosis to inhibit the development of postoperative cognitive dysfunction (Gu and Zhu, 2021). Decreased S-100β protein, neuron-specific enolase, and glial cell line-derived neurotrophic factor in the peripheral circulation are strongly associated with the development of POCD (McDonagh et al., 2010). These proteins may be modified and increased or decreased through the extracellular vesicle pathway to accelerate recovery from postoperative cognitive impairment. Extracellular vesicle-mediated circRNAs can also be involved in the onset and progression of neurological diseases (Chen et al., 2016). For example, the pathogenesis of POCD may be related to abnormal levels of exosome-delivered circRNA-089763 caused by perioperative stimuli.

Furthermore, elucidating the mechanisms by which postoperative extracellular vesicle changes control neuroinflammation is critical for developing therapeutic strategies targeting postoperative neurodegeneration (Prinz and Priller, 2014; Gao and Hong, 2008; Izquierdo-Altarejos et al., 2020). In this context, extracellular vesicles have emerged as key mediators mediating circulatory and central inflammatory changes, facilitating the transfer of inflammatory bioactive molecules between cells and the regulation of immune responses. Extracellular vesicles act as carriers of specific cargoes, including microRNAs, proteins, and lipids, and are capable of modulating peripheral and central immune responses (Vella et al., 2016; Gallego et al., 2022). For example, extracellular vesicles from activated microglia can exacerbate central inflammation by transferring pro-inflammatory molecules to neighboring cells, while other studies have shown that extracellular vesicles can transport inflammation-suppressing substances to control excessive immune responses (Izquierdo-Altarejos et al., 2023).

### Cell-free nanotherapeutic pathways

**FIGURE 2:**
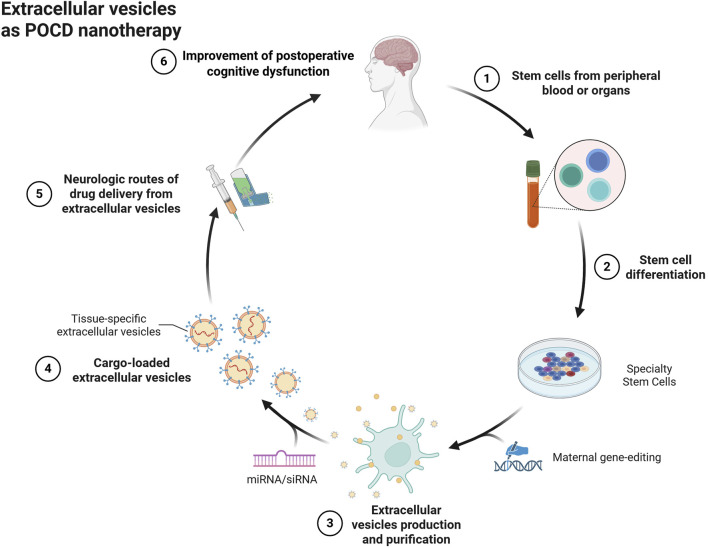
Extracellular vesicles as POCD nanotherapeutics.

For the treatment of POCD, extracellular vesicles of specialized cellular origin may be good cell-free nanotherapeutic avenues (Figure 2). Compared to liposomes, extracellular vesicles have excellent targeting and alteration of pathophysiological processes due to their specific membrane proteins and contained biomolecules, while their low immunogenicity and biohazardous nature may be of greater value for use in purely cell-based therapies. For example, MSCs are pluripotent cells with potential regenerative repair potential, mainly by secreting growth factors and cytokines for paracrine effects. Extracellular vesicles with cellular repair function are one of their important paracrine factors, which may be a potential cell-free nanotherapeutic for the treatment of CNS diseases. Recent reports suggest that extracellular vesicles can prevent neuroinflammatory properties in the center (Zhuang et al., 2011). For example, MSC-derived extracellular vesicles prevented postoperative hippocampal tissue damage and significantly reduced serum NSE and S100-β levels. Extracellular vesicles derived from human mesenchymal stem cells have been shown to have anti-inflammatory effects on microglia in perinatal brain injury (Thomi et al., 2019) and the number of activated inflammatory microglia was significantly reduced compared to the control group (Zhuang et al., 2011). Similar to this result, adipose stem cell-derived extracellular vesicles inhibited microglia activation and prevented neuroinflammation by inhibiting NF-κB and MAPK pathways (Yang et al., 2017). Extracellular vesicles of antler MSCs can help improve cognitive function in rats undergoing extracorporeal circulation surgery by mediating the TLR2/TLR4 signaling pathway, which may be due to the fact that extracellular vesicles of AMSCs inhibit neuronal apoptotic ability in rats undergoing extracorporeal circulation surgery. In fact, in addition to stem cell-derived extracellular vesicles, more cell-derived extracellular vesicles exist to improve postoperative cognitive dysfunction, such as plant-derived extracellular vesicles (Nemati et al., 2022). Ginseng-derived extracellular vesicles were found to have special proteins that nourish nerve tissues to repair damaged neurons (Li et al., 2023). In contrast, extracellular vesicles of poplar origin function to inhibit the systemic inflammatory response (Shao et al., 2023). This may also be a potential tool to improve postoperative cognitive dysfunction.

### Penetrating the blood-brain barrier to target cells

In fact, due to their phospholipid bilayer structure, extracellular vesicles can penetrate all current biological barriers, such as the blood-testis barrier, the blood-brain barrier, and the placental barrier. In the case of the blood-brain barrier, the mechanism by which extracellular vesicles cross the blood-brain barrier is through receptor-mediated endocytosis, e.g., extracellular vesicles derived from neural stem cells interact with brain endothelial cells *via* heparan sulfate proteoglycans and ultimately cross the blood-brain barrier through endocytosis (Yin et al., 2025). For the treatment of central diseases, the penetration rate of the blood-brain barrier of a drug is a key indicator for evaluating drug efficacy. This may not be necessary for extracellular vesicles, such as those administered intranasally, which can be rapidly transported to the mouse brain and taken up by microglia. It can also be engineered to modify its membrane proteins to enhance its central targeting ability. It has been found that receptor-mediated transcytosis is widespread at certain receptors (e.g., TfR, low-density lipoprotein receptor (LDLR), and insulin receptor (INSR)) on brain endothelial cells. Utilizing these receptor pathways, enhancement of extracellular vesicle penetration into the BBB can be designed. For example, extracellular vesicles harboring TfR ligands cross the BBB *via* receptor-mediated transcytosis (Wang et al., 2024). In addition, in clinical work, peripheral blood samples are easier to collect and accept than other samples such as cerebrospinal fluid and brain tissue, and extracellular vesicles in the extracorporeal circulation may be excellent diagnostic markers for central diseases. It has been found that brain tissue can release extracellular vesicles, which can carry non-coding RNAs (ncRNAs, microRNAs, circRNAs) and enter the circulation through the blood-brain barrier. Especially for circRNA, it is mainly transported in the form of extracellular vesicles and communicates between central and peripheral circulation through the blood-brain barrier. (Li et al., 2015). circRNA may play a key role in neurological disorders (Zhao et al., 2016; Sekar et al., 2018). For example, circRNA-associated ceRNA networks have been characterized in the susceptible brains of mice with accelerated aging, and these networks may influence the diagnosis and treatment of AD in the near future (Zhang et al., 2017). Furthermore, dysfunction of the extracellular vesicle-mediated circRNA-miRNA-mRNA regulatory system appears to represent an important aspect of epigenetic control of human CNS pathogenic genes. And POCD and AD have similar pathogenesis (Hu et al., 2010). Thus, the potential for the diagnostic and therapeutic role of extracellular vesicles in the treatment of central diseases is great.

### Communicating whole body holistic conditioning

Extracellular vesicles act as mediators for communication between the periphery and the center, and pathophysiological changes in the peripheral circulation can interact through extracellular vesicles. Extracellular vesicles play a key role in intercellular and interorgan communication and influence disease progression. Peripheral immune signaling can influence brain function through extracellular vesicle-mediated communication, which can affect barrier function and neuroinflammatory responses. Extracellular vesicles contain key mediators in the immune system (Budnik et al., 2016). These mediators activate the peripheral inflammatory response to induce the production of pro-inflammatory factors, which, after blood transport and penetration of the disrupted blood-brain barrier, can enter the center and exacerbate the neuroinflammatory process in neurological disorders. Second, proinflammatory factors can also be transported directly to the center by extracellular vesicles, inducing a central inflammatory response (Cabrera-Pastor, 2024). In addition, there is evidence that extracellular vesicles play a role in facilitating communication between the brain and adipose tissue. Adipose tissue produces many bioactive substances that are involved in interactions with peripheral organs and the central system through extracellular vesicles. Adipokines transported by extracellular vesicles modulate neuroinflammation and oxidative stress, which have been implicated in central nervous system disorders. In addition, adipose tissue is an important source of circulating noncoding RNAs, many of which are carried by extracellular vesicles and are involved in regulating homeostasis in the peripheral and central systems (Hajer et al., 2008). For chronic diseases resulting in systemic pathological microchanges can also be transmitted centrally *via* extracellular vesicles, inducing atrophy and aging of neural tissues, e.g., POCD. POCD is also an important comorbidity and complication of diabetes (Biessels and Whitmer, 2020). Diabetic patients are at a significantly increased risk of developing cognitive dysfunction after surgery (Thourani et al., 1999). This may be related to diabetes modulating cognitive function and changes in brain structure, such as diabetics having lower total brain volume, more infarcts, greater white matter high signal volume, and lower gray matter volume (Callisaya et al., 2019). In contrast, extracellular vesicles may be involved in inducing changes in the brain tissue of diabetic patients, possibly because they transmit signals of circulating chronic inflammation and abnormal glucose metabolism that lead to neurological tissue lesions (Wang M. et al., 2019). For example, glucagon signaling pathways have been shown to be associated with neuroinflammation, and these signals can also be transmitted by extracellular vesicles (van Harten et al., 2012; Gonçalves et al., 2016; van Praag et al., 2002; Zhang L. et al., 2023). And neuroinflammation has become an important cause of aging and cognitive decline. In addition, gut microbes play a role in the production of bioactive substances, such as extracellular vesicles (Mhanna et al., 2024; Zhai et al., 2024). These extracellular vesicles can enter the bloodstream and affect the central nervous system, subsequently affecting host cognition and behavior. The perioperative period is characterized by disturbances in the intestinal flora due to slow gastrointestinal motility and metabolic disturbances in the body, while the extracellular vesicles produced by the mother are altered, and these altered circulating extracellular vesicles ultimately affect central cognitive functions (Huang et al., 2025).

## Discussion and outlook

Perioperative neurocognitive dysfunction is a major problem affecting the health of the population, compromising postoperative recovery and increasing the financial burden on patients. As survival life expectancy increases, POCD is a growing public health problem due to the need for surgery in many frail older adults, with approximately 20%–50% of people over the age of 60 years developing POCD after major surgery (Avidan et al., 2017; Vlisides et al., 2019). The pathogenesis of POCD involves a variety of neurobiological processes, including cerebrovascular dysfunction, neural tissue damage, neuroinflammation, and brain tissue alterations (Vlisides et al., 2019). For example, preoperative dexamethasone administration before cardiac surgery significantly reduced the incidence of postoperative neurocognitive deficits in patients. The mechanism of this protective effect is most likely through the reduction of the neuroinflammatory response (Glumac et al., 2017). This emphasizes the key role of neuroinflammation in the development of cognitive impairment. Therefore, ways to reduce neuroinflammation may be the most currently recognized effective way to improve POCD. However, at present, relying solely on drugs such as compounds that are not suitable for most elderly patients who need prophylactic or anti-infective drugs such as dexamethasone and immunosuppressants may have numerous side effects. These medications also fail to meet the practical requirements of sustained postoperative slowing of the inflammatory state in order to effectively prevent POCD. As far as extracellular vesicles are concerned, the role of extracellular vesicles in slowing down inflammation is more modest (Noonin and Thongboonkerd, 2021). It is also more suitable for perioperative maintenance medication. First, for the process of postoperative peripheral inflammation inducing central inflammation, blocking the mediator function of extracellular vesicles or utilizing extracellular vesicles can suppress peripheral immune activation which is also the initial state (Arabpour et al., 2021). Second, interfering with inflammatory activation of microglia as well as promoting the release of anti-inflammatory extracellular vesicles from the hippocampus through specific modifications targeting extracellular vesicles in the center (Qin et al., 2024; Xin et al., 2017; Kong et al., 2024; Xin et al., 2022; Feng et al., 2017). Finally, the regenerative function exhibited by extracellular vesicles of special origin contributes to the repair of nerve tissue damage caused by neuroinflammation (Lang et al., 2023). All these modalities suggest the great potential of extracellular vesicles for the prevention and treatment of POCD. For molecules of the delivery signaling pathway of extracellular vesicles, a role involving gut-brain communication could be interesting. By utilizing extracellular vesicles released by the cells may help to improve intestinal motility while improving central cognitive function (Diaz-Garrido et al., 2021). In fact, in elderly patients, the postoperative period is often accompanied by slow intestinal motility leading to the accumulation of harmful substances (metabolic wastes released by bacteria) in the intestines and into the bloodstream affecting central cognitive functions (Kim et al., 2024). Currently, the diagnosis of POCD is still based on the description of the patient’s symptoms, assessment of mental status, and clinical behaviors, which are complex and take time to evaluate, especially in older patients with preoperative psychiatric disorders (Needham et al., 2017; Kapoor et al., 2019). This greatly delays POCD diagnosis and treatment. Recent studies have found that diseased tissue from the brain can release extracellular vesicles and can cross the blood-brain barrier into the circulation. These extracellular vesicles may be a new diagnostic marker for POCD (Li et al., 2018; Otero-Ortega et al., 2019). Currently there are few effective treatments for POCD, and improving postoperative pain is considered one of them, and opioids and NSAIDs are commonly used clinically to relieve pain caused by postoperative period (Ho et al., 2024). However, continued opioid use increases the risk of serious adverse effects, such as drug addiction, opioid tolerance, and opioid-induced pain hypersensitivity. It should be noted that certain miRNAs carried by extracellular vesicles have sustained analgesic effects (Hua et al., 2022). However, since miRNAs can act on different target genes and the expression of a gene can be regulated by multiple miRNAs. This suggests that interventions based on targeting the activity and/or treatment of individual genes have a limited effect. Thus, the use of multiple extracellular vesicles with analgesic effect miRNAs or the use of extracellular vesicles implanted with multiple miRNAs, *etc.*, may be considered. Indeed, the successive multiple injuries induced by surgery require a multipronged therapeutic approach to ameliorate cognitive deficits, which includes systemic modulation using extracellular vesicles as well as reduction of the neurotoxic effects of anesthetic drugs (Cui et al., 2018). Of course, whether the use of multiple extracellular vesicles poses biosafety or potential complications needs to be further explored, for example, most of the extracellular vesicles that are administered *via* blood vessels or orally are concentrated in the liver and kidneys, and it is still unknown whether the metabolism of these extracellular vesicles by the liver will impair liver and kidney functions. In fact, administration *via* intranasal or intrathecal administration may be an effective way to avoid the above or even to rapidly affect the center (Yang et al., 2020).

## Summarize

Extracellular vesicles as a potential tool for the treatment of postoperative cognitive dysfunction. Extracellular vesicles may be effective in preventing the extent of postoperative neurocognitive deficits and contribute to the treatment of postoperative neural tissue damage by interfering with pathological mechanisms of postoperative cognitive dysfunction, such as inhibiting inflammation and repairing nerve damage. In particular, RNAs transmitted by extracellular vesicles modulate the systemic neuroinflammatory cascade response, neuronal injury, and signaling communication in systemic microecology. This suggests that the role of extracellular vesicles in the pathogenesis associated with postoperative cognitive dysfunction could provide an avenue for their role as potential biomarkers and therapeutic targets for neurocognitive deficits induced by surgical or anesthetic drugs. Continuing to elucidate the exact pathomechanisms of POCD, studies on the mechanisms of reliable action of extracellular vesicles in postoperative cognitive impairment, and how to use extracellular vesicles for rapid central onset of action may be the future direction of research in postoperative cognitive impairment. Future work should focus on how to obtain extracellular vesicles that have a significant effect and are conveniently sourced. For the present, particular attention could be paid to changes in the state of extracellular vesicles and intravesicular biomolecules in the presence of postoperative cognitive deficits for early diagnosis and intervention of the disease.
